# Super Divya, an Interactive Digital Storytelling Instructional Comic Series to Sustain Facilitation Skills of Labor and Delivery Nurse Mentors in Bihar, India—A Pilot Study

**DOI:** 10.3390/ijerph19052675

**Published:** 2022-02-25

**Authors:** Anika Kalra, Nidhi Subramaniam, Ojungsangla Longkumer, Manju Siju, Liya Susan Jose, Rohit Srivastava, Sunny Lin, Seema Handu, Sudha Murugesan, Mikelle Lloyd, Solange Madriz, Alisa Jenny, Kevin Thorn, Kimberly Calkins, Heidi Breeze-Harris, Susanna R. Cohen, Rakesh Ghosh, Dilys Walker

**Affiliations:** 1 Institute for Global Health Sciences, University of California San Francisco, 550 16th Street, San Francisco, CA 94158, USA; anika.kalra@ucsf.edu (A.K.); sunny.lin@ucsf.edu (S.L.); solange.madriz@ucsf.edu (S.M.); alisa.jenny@ucsf.edu (A.J.); dilys.walker@ucsf.edu (D.W.); 2 PRONTO India Foundation, Lucknow 226001, Uttar Pradesh, India; nidhi.sub91@gmail.com; 3 PRONTO India Foundation, Patna 800025, Bihar, India; ojungsangla@gmail.com (O.L.); ansiju@gmail.com (M.S.); liyalisoos@gmail.com (L.S.J.); rh.srivastava@gmail.com (R.S.); seema@prontointernational.org (S.H.); 4 CARE India, Patna 800025, Bihar, India; smurugesan@careindia.org; 5 Department of OB/GYN, The University of Utah, 30 North 1900 East, Salt Lake City, UT 84132, USA; mikelle.lloyd@utah.edu (M.L.); susanna.cohen@hsc.utah.edu (S.R.C.); 6 NuggetHead Studioz, LLC, 1862 Gracie Road, Hernando, MS 38632, USA; kevin@nuggethead.net; 7 PRONTO International, 5419 Greenwood Ave N, Seattle, WA 98103, USA; kimberly@prontointernational.org (K.C.); heidi@prontointernational.org (H.B.-H.)

**Keywords:** low- and middle-income countries, low resource settings, virtual intervention, health providers, simulation, mentoring and training, instructional comic, digital storytelling

## Abstract

To improve the quality of intrapartum care in public health facilities of Bihar, India, a statewide quality improvement program was implemented. Nurses participated in simulation sessions to improve their clinical, teamwork, and communication skills. Nurse mentors, tasked with facilitating these sessions, received training in best practices. To support the mentors in the on-going facilitation of these trainings, we developed a digital, interactive, comic series starring “Super Divya”, a simulation facilitation superhero. The objective of these modules was to reinforce key concepts of simulation facilitation in a less formal and more engaging way than traditional didactic lessons. This virtual platform offers the flexibility to watch modules frequently and at preferred times. This pilot study involved 205 simulation educators who were sent one module at a time. Shortly before sending the first module, nurses completed a baseline knowledge survey, followed by brief surveys after each module to assess change in knowledge. Significant improvements in knowledge were observed across individual scores from baseline to post-survey. A majority found Super Divya modules to be acceptable and feasible to use as a learning tool. However, a few abstract concepts in the modules were not well-understood, suggesting that more needs to be done to communicate their core meaning of these concepts.

## 1. Introduction

The quality of obstetric and neonatal care in Bihar, India, continues to be challenged by a complex inter-play of factors. Bihar has a neonatal mortality rate and maternal mortality ratio of 27 per 1000 and 165 per 100,000 live births, respectively [1]. Despite having less than 10% of the population in the country, Bihar accounts for 15% of neonatal deaths [2,3,4]. Primary health centers (PHCs) are the location for births for the over 44 million women residing in rural areas. However, PHCs are ill-equipped, chronically short staffed, and lack access to specialists, including obstetricians and gynecologists [1,3,5]. Intrapartum care in these PHCs is primarily provided by nurses with little training and experience. To improve maternal and neonatal outcomes, CARE India, a non-governmental organization, in partnership with the state Government of Bihar, implemented a large-scale quality improvement nurse-mentoring initiative. In 2014, PRONTO International joined the initiative to incorporate highly realistic clinical simulations and team training [6,7]. In order to achieve change at the scale needed in Bihar, a large cadre of nurse mentor supervisors (NMS) and an even larger group of nurse mentors (NM) were trained to conduct simulations in situ at the facility, in addition to other mentoring activities. These facility level NM became simulation champions or simulation educators. Once trained, a new challenge arose—how could the gains in education be sustained in a cost-effective and resource efficient manner?

In 2019, the LINQED (Leading Innovation in Quality-of-Care Education Development) team came up with the innovative idea of developing a series of interactive virtual education comic modules known as “The Adventures of Super Divya”. The goal of these comic modules is to maintain and strengthen the simulation educator’s facilitation skills. Comics can communicate complex ideas about health as the medium conveys nuanced concepts, explores emotional and social issues, and allows readers to develop a level of empathy beyond what can be achieved through textbooks [8]. In formal health education settings, comics have been used among medical, nursing, and psychiatry students, to teach diverse topics from empathy to anatomy [9,10,11]. The Super Divya modules are intended to provide virtual support and continued education opportunities to NMS and NM by offering interactive and engaging learning sessions that are available on-demand and can be disseminated at a low cost.

The interactive comic modules were created using iterative human-centered design to ground the content in the lived reality of conducting simulation in PHCs in Bihar [12]. The development of the current modules started with a beta testing period, which included focus group discussions and a pre-pilot survey of a few NMS in May 2019 and in January 2020. Feedback from these exercises showed the potential for nurse’s engagement and learning. After integrating feedback from the design process and initial qualitative interviews, we created 10 short modules in both English and Hindi. The piloting of this series of modules coincided with the COVID-19 pandemic, thus increasing the urgency for hybrid and virtual training opportunities. This mid-line study reports on a pilot of the first six Super Divya comic modules with a group of NMS and NM. The objectives of this mid-line pilot study are to (1) assess the change in knowledge level between the baseline and the post-module surveys; (2) understand the specific reactions of the NMS and NM to each module; and (3) explore opinions on feasibility, adoptability, and acceptability of this innovative training tool.

## 2. Methods

### 2.1. Pilot Study Setting

A large statewide quality improvement nurse-mentoring program, known as “AMANAT”, was implemented in Bihar between 2015 and 2017, which included 320 basic emergency obstetric and newborn care and 22 comprehensive emergency obstetric and newborn care facilities. As part of the AMANAT program, facility-based nurses were trained by about 120 NMS to improve clinical and non-clinical skills (i.e., teamwork and communication, and facilitation), along with bedside mentoring, demonstration of clinical procedures, and didactic lessons. During the AMANAT program, a pair of NMS spent one week per month training labor and delivery care nurses in each facility for eight consecutive months. Specific details of the program have been published elsewhere [13,14,15]. After the successful completion of the AMANAT program, in 2018, a modified version of the program known as “AMANAT Jyoti” was launched in 330 Basic Emergency Obstetric and Newborn Care and 35 Emergency Obstetric and Newborn Care facilities with an emphasis on sustainability and transition to management by the state Government of Bihar [16]. In the AMANAT Jyoti program, a training cascade was developed, where NMS were trained first before training two nurses (NM) selected from each facility to become facility-based mentors with training in simulation, among other topics. These NM are tasked with facilitating simulation and other mentoring activities in their respective facilities, with assistance from the NMS. A total of about 150 NMS and 1000 NM have been trained since 2018.

### 2.2. Super Divya Intervention

The Super Divya comic series focuses on reinforcing educator skills that NMS and NM need to effectively train others, using simulations. The Super Divya modules include an interactive story with two main characters: Divya, who has made it her life mission to spread the magic of facilitated simulation, and the antagonist Professor Agni, whose goal is to derail Divya’s efforts by distracting simulation educators and participants from engaging in meaningful simulation experiences. The storyline starts by portraying Professor Agni as a friend of Divya, but her first simulation experience was unpleasant, which made her feel unsafe about learning through simulation and she developed a repulsion towards it. Professor Agni and her schemes are meant to represent real life challenges that simulation educators face in their daily work. Divya displays strong leadership, effective facilitation, and communication skills to overcome every barrier encountered while conducting simulations in her facility. The comic images are fun, engaging, and reflective of the reality in the facilities in Bihar.

**Figure 1:**
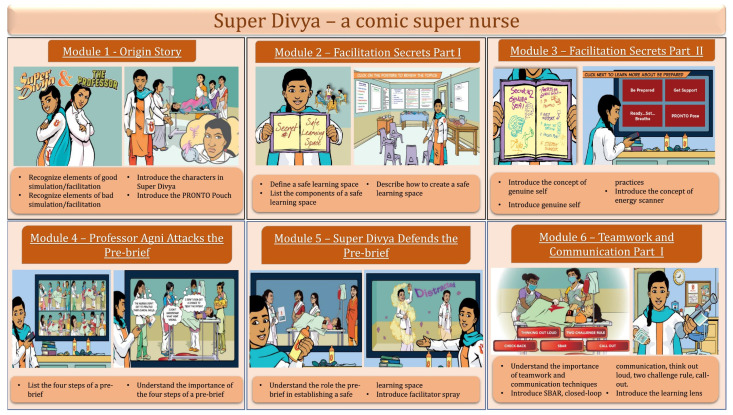
Description and the learning objectives of Super Divya modules 1 through 6.

Along with the storyline, the modules contain interactive components to keep nurses actively engaged in the learning process. Modules emphasize the skills needed to create a safe learning environment before conducting simulation or team training activities. They allow participants to learn and review topics in their own time by using various learning strategies and bringing the “feeling” of in-person training to the virtual space. Ten comic modules have been planned targeting both NMS and NM. The first six modules have been developed in both English and Hindi by the Lift Simulation Design Lab at the University of Utah using iterative techniques with feedback from nurses and translation by team members in India. Each Super Divya module is designed to focus on a specific simulation facilitation topic and has pre-defined learning objectives such as defining a safe learning space, listing the elements of a pre-brief, and introducing communication techniques (Figure 1). The Hindi version of the modules were sent via WhatsApp to NMS and NM working in PHCs, district hospitals, sub-divisional hospitals and referral hospitals in Bihar. Supplementary Figure S1 presents a timeline of the module distribution.

### 2.3. Survey Description

Bilingual (English and Hindi) online surveys were designed and distributed using a web-based platform, Qualtrics (Qualtrics, Provo, UT, USA). Before distributing Super Divya module 1, participants were sent a baseline survey that contained 20 true/false questions covering the objectives of all 10 modules (Supplementary Table S1). Participants received a post-module survey after they watched each module. The post surveys contained two questions from the baseline survey, and 2–5 additional questions based on the specific topics covered in the module (Supplementary Table S1). Nurses were able to take the surveys on their mobile phones, tablets, or computers. All questions were mandatory and responses included true/false, multiple choice, or selecting all that apply. Correct answer to each question were displayed after a survey was submitted, except for the baseline survey. All post-modules surveys contained a “reaction” question using emojis (happy, love, neutral, tired, and sick) to understand the nurse’s feelings after watching each module.

To measure mid-line feasibility, adoptability, and acceptability, we included additional questions in the post-module 5 survey (Supplementary Table S1). These questions were based on a Likert scale of “not at all”, “a little bit”, “a lot”, and “do not know”. NMS received a few additional questions on adoptability to understand their opinions on the potential for the adoption of Super Divya for the NM.

### 2.4. Sample Selection and Personnel

Super Divya was piloted with all NMS in the program. NMS were nursing professionals in a mentoring role with a Bachelor’s degree or higher, who were hired or contracted by CARE India. NM were convenience sampled considering recommendations by CARE India, based on the likelihood of their active participation in this novel intervention. NM are government employees who were either staff nurses or auxiliary nurse midwives. Staff nurses had a diploma in general nursing and midwifery, which is a three-year course in basic nursing that can be undertaken after completing high school. General nursing and midwifery train to conduct deliveries and manage obstetric complications. An auxiliary nursing midwifery qualification is a two-year diploma that can be undertaken after completing high school, and it trains individuals to be multipurpose community health workers. In India, the auxiliary nurse midwives diploma program includes course work, but little practical training in managing deliveries. In PHCs of Bihar, auxiliary nurse midwives have largely taken over the responsibility of conducting deliveries, as staff nurses are scarce [17,18].

### 2.5. Statistical Analysis

The analysis of the mid-line data included two approaches. In the first approach, we matched participants’ responses from baseline to post-module and estimated the proportion of correct responses per respondent at baseline and in the post-module survey. The denominator was the total number of survey questions. For example, if there were 12 total questions and Participant A answered six of them correctly, the score for participant A was 50%, hereafter referred to as “individual scores”. We reported individual scores in four categories: 100%, 99–80%, 79–60% and below 60%. Additionally, we also calculated the average of the individual scores across all participants.

In the second approach, we calculated the proportion of all participants that answered each question correctly. In other words, the denominator was total respondents. For example, if 10 of the total 100 respondents answered question one correctly, the group achievement on this question was 10%.

In order to make meaningful comparisons between baseline and post-module surveys in this mid-line analysis, we compared the matched performance on the 12 baseline questions that were repeated in the six post-module surveys. The baseline survey had additional questions beyond module 6, which were excluded from this analysis, for meaningful comparison. Additionally, we only included participants for whom we could match responses starting from baseline to module 6. We conducted McNemar’s test or McNemar’s exact test to examine statistical significance between paired proportions calculated from questions that were common between baseline and post-module surveys.

The question about respondent’s reactions after watching the module were presented as a proportion of the total respondents. Likewise, the series of questions related to feasibility, adoptability, and acceptability were presented as proportions of the total respondents. Analyses were conducted and reported separately for NMS and NM. We used Excel and R for the analysis and reported using the SQUIRE guidelines recommended for the quality improvement interventions [19].

### 2.6. Ethical Considerations

This study was approved by the Institutional Review Board of the University of California, San Francisco (IRB Ref No. 19-29715). In India, ethical approval was obtained from the All India Institute of Medical Sciences, Patna (Ref No. AIIMS/Pat/IEC/2020/700). Prior to participation in the Super Divya pilot, informed verbal consent was obtained from each participant. Additionally, all data were saved on password protected and encrypted computers provided by the University of California, San Francisco.

## 3. Results

A total of 105 NMS and 148 NM received the modules. After cleaning the data, 75 NMS and 130 NM were matched across baseline and all six post-module surveys. The vast majority of NMS had a Bachelor’s degree or higher, while almost all NM had a general nursing and midwifery or an auxiliary nursing and midwifery training (Table 1). About 68% of NMS and 92% of NM had participated either in the AMANAT or AMANAT Jyoti programs. All NMS and three-fourths of NM were trained by PRONTO International on facilitating simulations and teamwork and communication activities (either through in-person, hybrid, or virtual trainings). A little less than three-quarters of NMS and half of NM facilitated 1 simulation or more per month (Table 1).

**Figure 2:**
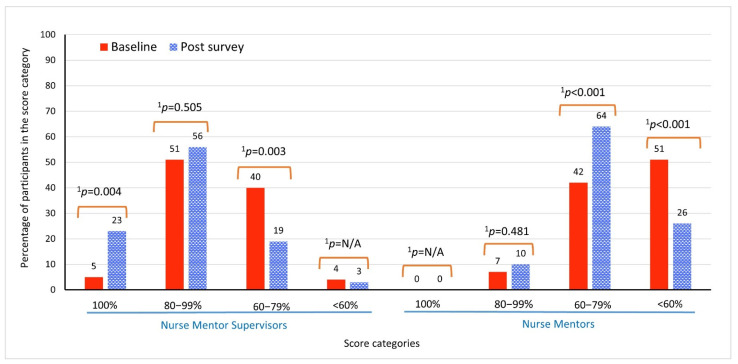
Performance of nurse mentor supervisors (NMS) (*n* = 75) and nurse mentors (NM) (*n* = 130) on the common set of 12 Super Divya questions that were asked both at baseline and in the post-module surveys. ^1^ *p*-values are from McNemar’s or McNemar’s Exact Test, as relevant. We did not report a *p*-value if it was 1 or if it could not be calculated because of 0 counts. Note: Among the NMS, the lowest score was 50% at baseline and 50% in the post-module surveys. Among the NM, the lowest score was 42% at baseline and 33% in the post-module surveys.

The results showed that individual scores improved when the performance over the 12 common questions, included both at baseline and post surveys, were examined in aggregate. The results for the NMS showed an upward trend from the second and third highest score categories at baseline to the topmost and second highest categories in the post-module surveys. For example, about 5% of NMS achieved 100% scores at baseline, whereas in the post-module surveys, 23% scored 100% (Figure 2). The percentage of NMS in the 80−99% category increased from 51% at baseline to 56% in the post-module surveys, those in the 60−79% category decreased from 40% at baseline to 19% at post-module surveys, and a small percentage that scored below 60% remained unchanged. There was a similar trend from low to high score categories among NM. None of the NM scored 100% at baseline or in the post-module surveys. While the percentage of NM in the 80−99% score category increased marginally, the largest improvement was observed in the 60−79% category, from 42% at baseline to 64% in the post-module surveys (Figure 2). The percentage of NM who scored below 60% decreased from 51% at baseline to 26% in the post-module surveys.

Correct responses to each question were also examined at baseline and in the post-module surveys. Comparing baseline with the post survey for the module 1 questions, NMS demonstrated a small improvement, but NM did considerably better (Table 2). Regardless, almost a third from both cadres were unaware that a facilitator does not have to know all the answers to participants’ questions, even after watching the module. For module 2, the baseline and post-module scores were very close to 100% for both cadres. Module 3 introduced two relatively new concepts—genuine self and energy scanner. On both of these topics, NMS performed better than NM. Furthermore, the post survey results suggest that the concept of the genuine self appears to be better understood than the concept of energy scanner, by both cadres (Table 2). For module 4, the scores of the NMS were largely unchanged for both the questions, but their baseline levels were about 90% or higher. However, the NM increased their scores substantially on the topic of pre-brief (Table 2). For module 5, more than half of the NMS and NM thought that facilitators need to ignore their own emotions to successfully conduct a simulation training, and the modules appear to have led to a modest increase in knowledge (Table 2). Similarly, for module 6, about half of NMS and 90% of NM thought that clinical knowledge is about correct behaviors, which the module helped them understand correctly, as evidenced by the post survey results (Table 2). To navigate and watch a comic module, click on the hyperlinks inserted in the first column in Table 2.

**Figure 3:**
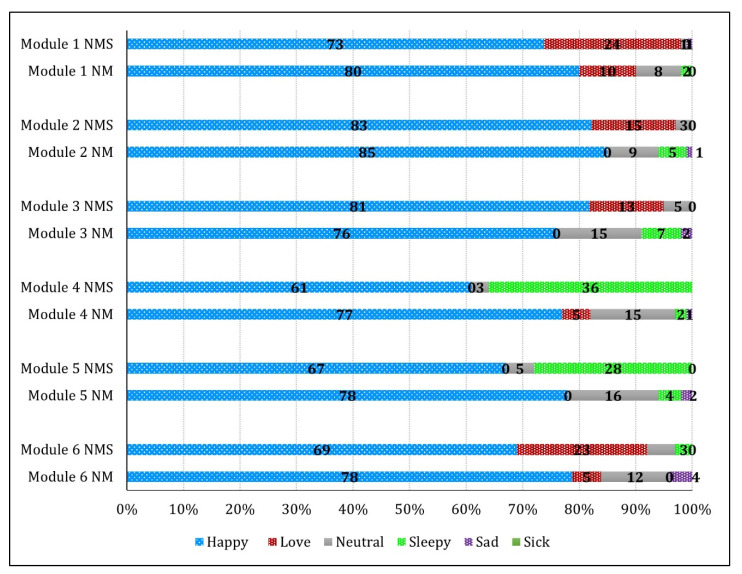
Reactions of nurse mentor supervisors (NMS) (*n* = 75) and nurse mentors (NM) (*n* = 130) after watching Super Divya modules 1 to 6 ^1^. ^1^ Percentage of NMS and NM.

Figure 3 shows the reactions of the NMS and NM after watching the modules. The vast majority of NMS and NM were happy after watching the modules, although a percentage of NM had no reaction. Some NMS loved watching modules 1, 2, and 3, and 28-36% registered a sleepy reaction after watching modules 4 and 5, whereas 15−16% of NM had a neutral reaction.

**Figure 4:**
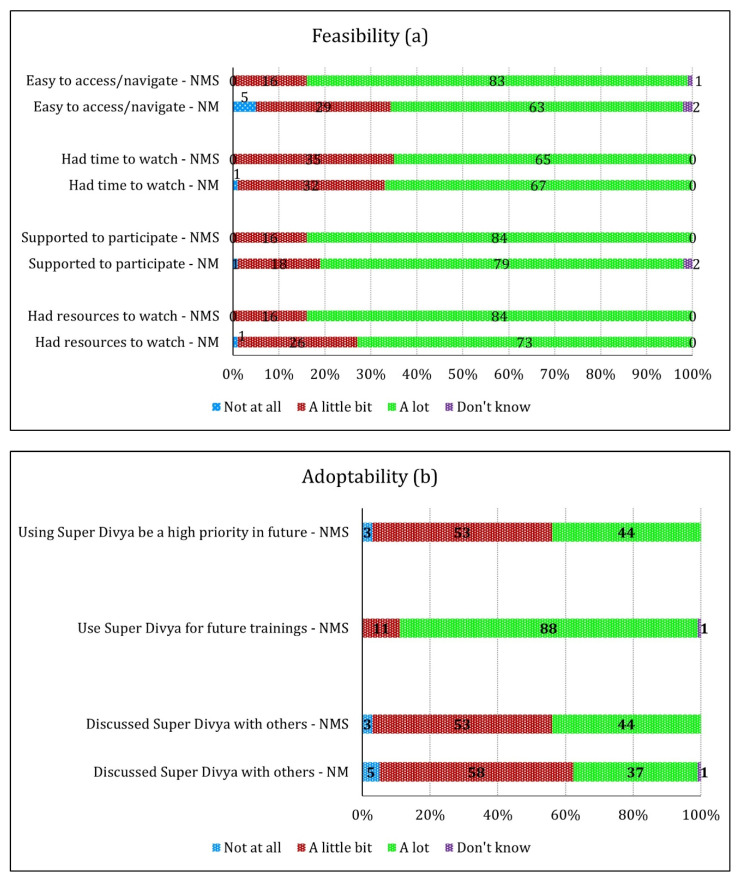

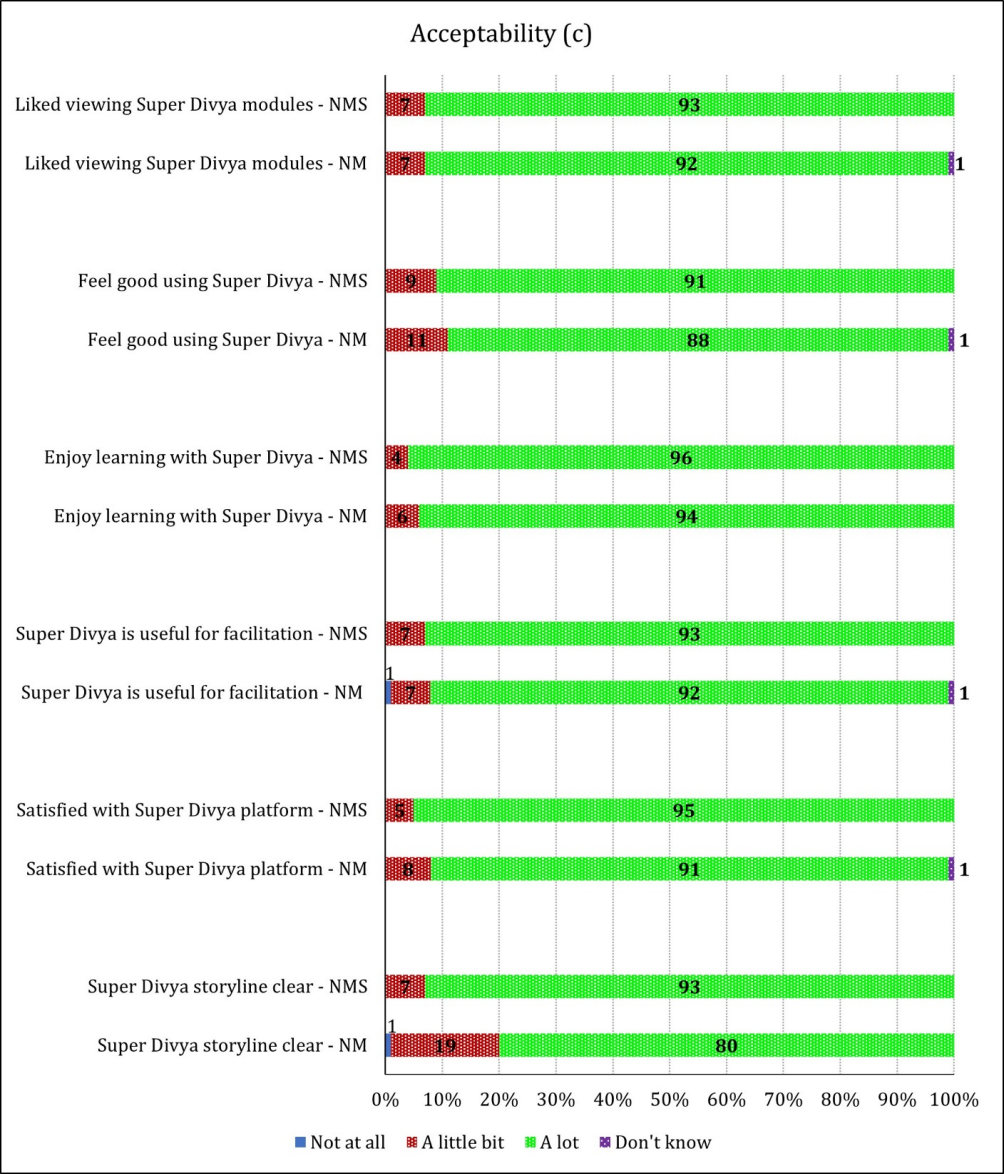
Feasibility (**a**), adoptability (**b**), and acceptability (**c**) of Super Divya as a comic instructional module for nurse mentor supervisors (NMS) (*n* = 75) and nurse mentors (NM) (*n* = 130) ^1^. ^1^ Percentage of NMS and NM.

The results for the feasibility, adoptability, and acceptability of Super Divya are shown in Figure 4a–c. Feasibility was measured using four items on a four-point Likert scale. Overall, 65–84% of NMS and 63–79% of NM reported “a lot” for easy to access, time to watch, supporting participation in the intervention, and had resources to watch the modules (Figure 4a). Adoptability was measured using one to three items on the same four-point Likert scale. The results show 44–88% of NMS thought that adopting Super Divya should be a high priority, should be used in trainings, and that they would discuss it with other nurses (Figure 4b). Over 90% of NM reported that they had discussed Super Divya with others at least “a little bit” or “a lot” (Figure 4b). Acceptability was measured similarly using six items. Between 91–95% of NMS and 80–94% of NM responded with “a lot” for all of the six items, which covered the following: liked viewing the modules, felt good about using the modules, enjoyed learning from the modules, thought the skills presented are useful for training, felt satisfied with this new platform, and thought the storyline was clear (Figure 4c). Notably, 1% of NM said Super Divya was “not at all” useful for facilitation, and that the storyline was “not at all” clear (Figure 4c).

## 4. Discussion

In this mid-line pilot study, we explored the use of the Super Divya comic series on sustaining and improving simulation and team training knowledge for NMS and NM simulation educators in Bihar, India. Individual scores tended to increase from baseline to post-module surveys. These findings suggest that the comic modules appear to have been successful in increasing the knowledge of NMS and NM on concepts important to successfully conduct simulation training (i.e., creating a safe learning environment and conducting a pre-brief). Substantial improvements were observed on several topics that were low scoring at baseline. The comic modules also attempted to introduce novel concepts, such as the concept of being centered and focused during simulation, which was conveyed through the metaphor of an “energy scanner”. For these more complex and new topics, the observed increase in knowledge was limited. Among the six modules, both cadres of simulation educators performed the worst on module 5, which is about understanding how emotions and attitudes as a facilitator can influence the ability to teach others. The module emphasizes the need to “name” emotions in order to work past them. This is an abstract concept that we believe is new to both cadres.

When comparing baseline to post-module survey scores by individual questions, the proportion of participants with the correct response either improved relative to baseline, or were close to 100% at baseline, thus leaving little room for improvement. Of particular note is module 2, which is about safe learning spaces—there was no significant improvement for either question, but both had very high scores at baseline (~98%) and in the post-module survey. The concept of a safe learning space has been heavily emphasized in all of PRONTO International’s trainings in Bihar. Nevertheless, the comic series likely served as short refresher trainings during the pandemic when there were practically no in-person activities to facilitate.

The modules on origin story, facilitation secrets, and teamwork and communication were the most popular ones (over 90% felt happy or loved it) among NMS, while the modules on origin story, facilitation secrets, pre-brief, and teamwork and communication were most popular (over 80% reportedly felt happy or loved it) among NM. Modules that focused on pre-brief and facilitation secrets received a larger proportion of sleepy and neutral reactions by NMS and NM, respectively, in comparison to other modules. Additionally, differences in reactions to modules 4 and 5 relating to the pre-brief by the two cadres suggest that tailoring modules to specific level of staff (as different levels have different trainings) may increase the appeal of Super Divya.

Given that a vast majority of simulation educators received prior PRONTO simulation facilitation training, there could be several likely reasons for the improvement in knowledge, including exposure to Super Divya modules. Nursing education is one likely factor that may impact performance. NMS hold more advanced degrees than NM, and their performance overall and on individual questions was consistently better than NM. Furthermore, the findings suggest that simulation educators performed worst on questions relating to facilitator knowledge, genuine self, and energy scanner, which are figurative concepts. These types of concepts need to be further explored to delineate if the simulation educators do not understand the underlying concepts or if they are aware of the concepts but are unable to connect these metaphors to the concepts. It may also be that the questions developed to measure these concepts were too abstract themselves, thus causing further confusion.

This mid-line pilot study also aimed to understand if the innovative Super Divya comic series is feasible to implement, adoptable in the local context and if it is acceptable to the simulation educators. Overall, our results show that the vast majority of NMS and NM felt that Super Divya modules were feasible, adoptable, and acceptable to use. Between 60% and 85% of the simulation educators reportedly felt happy after watching the modules, demonstrating that Super Divya was well received. Over 80% of NMS and 60% of NM stated that the modules were easy to access and navigate. They felt supported in the program and had enough time and internet access to watch the modules. The responses suggest that both cadres enjoyed learning and watching the modules and could use the skills presented to facilitate simulation training in their facilities. Additionally, NMS and NM were able to relate to the scenarios in the modules, making the modules realistic. These findings are encouraging and indicate that the Super Divya comic series could potentially be a supportive learning tool to reinforce simulation education.

To the best of our knowledge, there is no directly comparable literature on the use of innovative comic education modules to improve the knowledge and engagement of simulation educators in a low resource setting. However, studies have demonstrated the effectiveness of virtual modules and virtual simulation in improving knowledge on maternal and neonatal care. In particular, the use of digital simulations and tools in Canada was found to improve provider’s knowledge on neonatal resuscitation from pre- to post-module, and was retained two months post-module [20,21,22,23]. A literature review showed knowledge improves with the use of simulation-based educational games for neonatal resuscitation [24]. Furthermore, video debriefing as a supplement to didactic training improved the knowledge and retention of skilled birth attendants in Uganda [25]. These prior studies, although not directly comparable to the unique Super Divya comic series, demonstrate the potential of both comics and video-based training to engage simulation educators and sustain acquired knowledge over time. Moreover, video-based training modalities have significant advantages, especially during the COVID-19 pandemic. Our study shows that during a pandemic, Super Divya modules can be used to provide virtual support and continued education to NMS and NM by offering interactive and engaging learning sessions that are available on demand. Despite not being able to undertake in-person trainings, nurses could watch training modules at any time on their mobile devices. The Super Divya comic series available through a virtual platform has the potential to be a valuable resource for boosting the knowledge, facilitation skills, and self-confidence of simulation educators as the need for hybrid and virtual training increases globally.

This study was strengthened by the creativity and innovation of Super Divya. As each module was unique with different learning objectives, the attractive graphics and interesting storylines helped simulation educators feel connected to their real-life experiences. Regular inputs in the development process from master mentors who were once NMS and their contribution in translating modules into the local language (Hindi) made these modules culturally appropriate and relatable to the simulation educators in Bihar. However, there were some barriers, including difficulties accessing the modules due to connectivity issues and registering responses to the online surveys in a small number of participants. To alleviate these problems, CARE-India provided nurses partaking in the implementation with data SIM cards. It is also important to recognize that the survey tools may have introduced bias, as the questions were not tested for validity, and surveys with more or less questions than the standard five may have caused stress or confusion. Additionally, we did not have a way to ensure that nurses responded to post-module surveys only after viewing the modules; thus, some nurses may have responded to surveys without watching Super Divya modules, which may have skewed the validity of our results. It is also possible that participation in previous PRONTO and AMANAT trainings and years of experience as a simulation educator influenced the baseline and post-module knowledge of the simulation educators. It is challenging to attribute learning from these different trainings that happened over several years. Finally, we faced challenges with the roll out of Super Divya modules. We initially distributed the modules to 105 NMS and 148 NM. Of these, 75 NMS and 130 NM were retained, as some were reassigned to COVID-19 duty or transferred to other departments within the same or another facility. This loss to follow-up may have introduced bias in our results. Due to constraints beyond the control of the study team, such as new hires and transfers of government staff, many NMS moved on from the program. As a result, moving forward, we will lose about one-third of NMS and a relatively small percentage of NM. In this mid-line study, we present the results of the implementation of modules 1–6. Subsequent modules will be piloted on fewer simulation educators and an end-line survey will provide more insight on the retention of knowledge over time.

## 5. Conclusions

Overall, the Super Divya modules appear to have helped improve knowledge for two cadres of simulation educators in Bihar. The results suggest that these modules were received as feasible, adoptable, and acceptable training tools. Several gaps have remained that point to the need for the additional reinforcement of more complex and new concepts. Nevertheless, the results from this pilot study are encouraging, especially during a pandemic when virtual education is necessary. These findings suggest the Super Divya comic series as an innovative training tool that can be used for a wider roll out to all simulation educators in the state who have been trained as simulation educators in order to improve the quality of obstetric and neonatal care.

## Figures and Tables

**Table 1:** Characteristics of the nurse mentor supervisors and nurse mentors, the participants of the Super Divya modules and surveys implemented from May to September 2021.

	Nurse Mentor Supervisors (*n* = 75)	Nurse Mentors (*n* = 130)
	% (*n*)	% (*n*)
Qualification		
General nursing and midwifery	N/A	83 (108)
Auxiliary nursing and midwifery	N/A	15 (19)
Post diploma in nursing	8 (6)	N/A
Bachelor’s in nursing	80 (60)	2 (2)
Master’s in nursing	12 (9)	0 (0)
Not reported	0 (0)	1 (1)
Participated in the AMANAT or AMANAT Jyoti Programs		
Yes	68 (51)	92 (119)
No	32 (24)	8 (11)
Previously trained by PRONTO		
Yes	100 (75)	77 (100)
No	0 (0)	23 (30)
Number of simulations conducted monthly prior to COVID-19 interruptions		
0	28 (21)	52 (68)
1–3	59 (44)	45 (59)
4–6	11 (8)	1 (1)
7–9	0 (0)	0 (0)
10 or more	3 (2) ^1^	2 (2)

^1^ One NMS wrote, ‘Not in every month’ in this option.

**Table 2:** Performance on the 12 individual questions that were asked both at baseline and in the post surveys.

Module	Survey Question	Staff Category	Baseline Score % (*n*)	Post Survey Score % (*n*)	*p*-Value ^2^
Module 1—Origin Story	A facilitator knows all the answer to participants’ questions.	NMS ^1^	51 (38)	59 (44)	0.239
		NM ^1^	15 (19)	65 (84)	<0.001
	A facilitator should stop the simulation when a nurse misses a clinical management step.	NMS	91 (68)	92 (69)	N/A ^3^
		NM	73 (95)	92 (119)	<0.001
Module 2—Facilitation Secrets Part I	Greeting nurses when they arrive at the training is an important step in creating safe learning space.	NMS	99 (74)	100 (75)	N/A ^4^
		NM	98 (127)	98 (127)	N/A ^3^
	In a safe learning space, nurses feel supported and open to learning	NMS	100 (75)	100 (75)	N/A ^4^
		NM	99 (129)	99 (129)	N/A ^3^
Module 3—Facilitation Secrets Part II	To bring my genuine self, I need to avoid challenging situations.	NMS	91 (68)	93 (73)	0.727
		NM	68 (88)	68 (88)	N/A ^3^
	The energy scanner is a tool that Super Divya uses to measure the energy of others in the room.	NMS	53 (40)	77 (58)	0.001
		NM	30 (39)	48 (63)	0.002
Module 4—Professor Agni Attacks the Pre-brief	During the pre-brief, the facilitator should remind participants how to manage medical complications.	NMS	84 (63)	89 (67)	0.424
		NM	27 (35)	86 (112)	<0.001
	During the pre-brief, the facilitator should allow providers time to review the simulation area.	NMS	92 (69)	95 (71)	0.727
		NM	94 (122)	98 (127)	0.227
Module 5—Super Divya Defends the Pre-brief	A genuine self-practice can help a facilitator calm her mind to feel centered.	NMS	97 (73)	96 (72)	N/A ^3^
		NM	97 (126)	95 (124)	0.754
	For a successful simulation training, the facilitators need to ignore their own emotion.	NMS	47 (35)	55 (41)	0.304
		NM	42 (54)	65 (84)	0.002
Module 6—Teamwork and communication	Clinical knowledge is a behavioral objective.	NMS	51 (38)	81 (61)	<0.001
		NM	14 (18)	51 (66)	<0.001
	If there are communication issues in the simulation, the facilitator should bring them up in the debrief.	NMS	97 (73)	100 (75)	N/A ^4^
		NM	98 (127)	93 (121)	0.227

^1^ NMS refers to nurse mentor supervisor (75) and NM refers to nurse mentors (130); ^2^ *p*-value from McNemar’s or McNemar’s Exact Test, as relevant; ^3^ *p*-value equal to 1; ^4^ *p*-value not generated due to 100% score.

## Data Availability

De-identified data pertaining to this manuscript will be made available upon reasonable request to the corresponding author. The data cannot be made publicly available due to regulations of the State Government of Bihar.

## Supplementary Materials

The following supporting information can be downloaded at: https://www.mdpi.com/article/10.3390/ijerph19052675/s1. Supplementary Figure S1: Super Divya module distribution timeline. Supplementary Table S1: Super Divya (SD) module (M) knowledge survey tools.
