# Supplementary material for: Super Divya, an Interactive Digital Storytelling Instructional Comic Series to Sustain Facilitation Skills of Labor and Delivery Nurse Mentors in Bihar, India—A Pilot Study
Source: Int J Environ Res Public Health. 2022 Feb 25;19(5):2675. doi: 10.3390/ijerph19052675 (PMC8910046; doi:10.3390/ijerph19052675)
Supplement: Supplementary file 1 [file ijerph-19-02675-s001.zip › ijerph-1569256-supplementary.pdf]

S1-Super Divya Manuscript Supplementary Information

Super Divya, an Interactive Digital Storytelling Instructional Comic Series to Sustain Facilitation Skills of Labor and Delivery Nurse Mentors in Bihar, India—A Pilot Study

Anika Kalra [1], Nidhi Subramaniam [2], Ojungsangla Longkumer [3], Manju Siju [3], Liya Susan Jose [3], Rohit Srivastava [3], Sunny Lin [1], Seema Handu [3], Sudha Murugesan [4], Mikelle Lloyd [5], Solange Madriz [1], Alisa Jenny [1], Kevin Thorn [6], Kimberly Calkins [7], Heidi Breeze-Harris [7], Susanna R. Cohen [5], Rakesh Ghosh [1,*] and Dilys Walker [1]

SUPPLEMENTARY INFORMATION

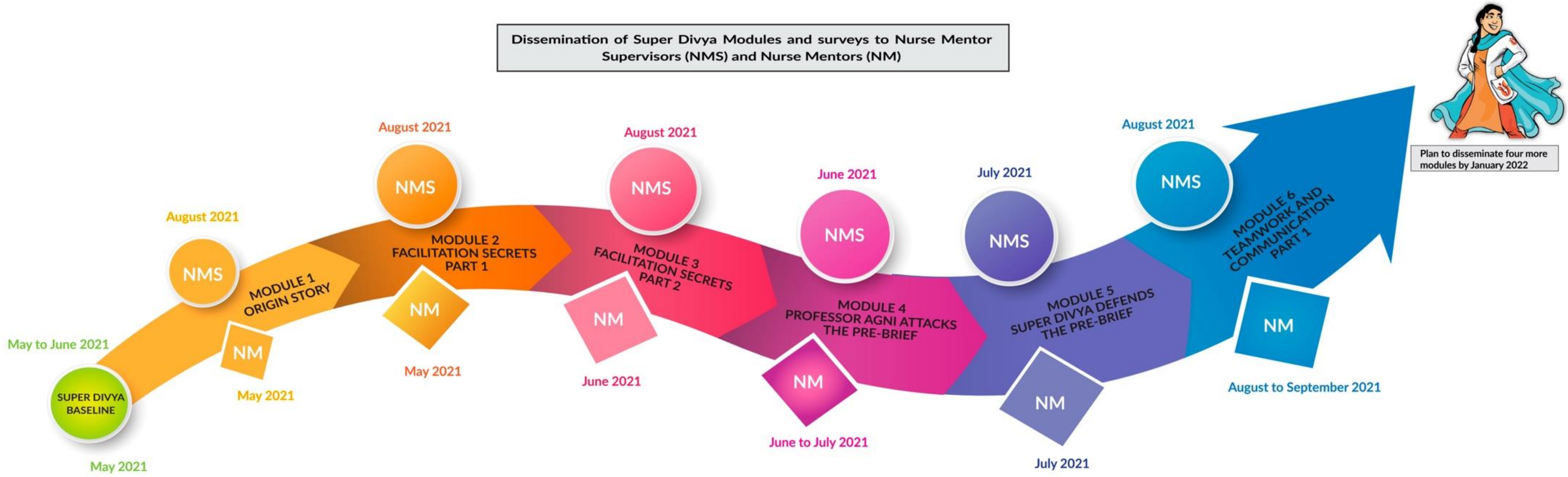

Supplementary Figure S1. Super Divya module distribution timeline.

Supplementary Table S1: Super Divya (SD) Module (M) Knowledge Survey Tools.

Baseline Survey Tool			
**Topics**	**Knowledge Questions**	**Choices**	**Correct Answer**
**SD M1: Origin**	1. A facilitator knows all the answer to participants' questions.	True/False	False
	2. A facilitator should stop the simulation when a nurse misses a clinical management step.	True/False	False
**SD M2: Facilitation secrets part 1**	3. Greeting nurses when they arrive at the training is an important step in creating safe learning space.	True/False	True
	4. In a safe learning space nurses feel supported and open to learning.	True/False	True
**SD M3: Facilitation secrets part 2**	5. To bring my genuine self, I need to avoid challenging situations.	True/False	False
	6. The energy scanner is a tool that Super Divya uses to measure the energy of others in the room.	True/False	False
**SD M4: Professor Agni attacks the Pre-brief**	7. During the pre-brief, the facilitator should remind participants how to manage medical complications.	True/False	False
	8. During the pre-brief, the facilitator should allow providers time to review the simulation area.	True/False	True
**SD M5: Super Divya defends the Pre-brief**	9. A genuine self-practice can help a facilitator calm her mind to feel centered.	True/False	True
	10. For a successful simulation training, the facilitators need to ignore their own emotions.	True/False	False
**SD M6: Teamwork and communication part 1**	11. Clinical knowledge is a behavioral objective.	True/False	False
	12. If there are communication issues in the simulation, the facilitator should bring them up in the debrief.	True/False	True
**SD M7: Teamwork and communication part 2**	13. An SBAR is given when a new nurse or doctor enters the clinical scenario to provide details of the patient's medical history.	True/False	False
	14. Paying attention during the simulation for examples of the communication techniques can help me prepare for the debrief.	True/False	True
	15. Empathy means to understand one's and other's feelings.	True/False	True

**SD M8: Introducing the Debrief**	16. One of the goals of the debrief huddle is to decide which nurses should speak up first.	True/False	False
**SD M9: Super Divya decodes the Debrief**	17. The debrief has 3 phases: description, recommendation, and application.	True/False	False
	18. Open-ended questions are helpful in the debrief to understand the nurses' thoughts and feelings.	True/False	True
**SD M10: The Epic Battle over the Debrief**	19. When participants show disinterest during the debrief, the facilitator should ignore them.	True/False	False
	20. If everything went well during the simulation, there's no need to have a debrief.	True/False	False
**Module 1 Survey Tool: Origin Story**			
**S. No.**	**Knowledge Questions**	**Choices**	**Correct Answer**
**1**	A facilitator makes everyone feel comfortable and welcome.	True/False	True
**2**	A facilitator knows all the answers to participants questions.	True/False	False
**3**	A facilitator encourages participants to learn from errors made in simulation.	True/False	True
**4**	A facilitator should stop the simulation when a participant makes a clinical error.	True/False	False
**5**	A facilitator points out only errors and ignores the good things.	True/False	False
**Module 2 Survey Tool: Facilitation secrets part 1**			
**S. No**	**Knowledge Questions**	**Choices**	**Correct Answer**
**1**	Greeting nurses when they arrive at the training is an important step in creating safe learning.	True/False	True
**2**	Greeting nurses when they arrive at the training is an important step in creating safe learning.	True/False	False
**3**	In a safe learning space nurses feel supported and open to learning.	True/False	True
**4**	Super Divya suggests to have a notebook to write down observations and questions during the simulation.	True/False	True
**5**	To create a safe learning space, the facilitator should locate and dispose the containers of hazardous biomedical waste for all participants.	True/False	False

Module 3 Survey Tool: Facilitation secrets part 2			
**S. No.**	**Knowledge Questions**	**Choices**	**Correct Answer**
**1**	To bring my genuine self, I need to avoid challenging situations.	True/False	False
**2**	A facilitator can influence how participants feel during the simulation.	True/False	True
**3**	According to Super Divya, the most important thing a facilitator brings to a simulation is her authority.	True/False	False
**4**	The concept of the genuine self means to work independently and solve my own problems.	True/False	False
**5**	The "Genuine Self" includes the facilitator's listening skills, empathy, compassion and curiosity.	True/False	True
**6**	The energy scanner is a tool that Super Divya uses to measure the energy of others in the room.	True/False	False
**7**	To bring my genuine self to a training, I need to know the correct answer for all the questions the nurses ask.	True/False	False
**Module 4 Survey Tool: Professor Agni attacks the Pre-brief**			
**S. No.**	**Knowledge Questions**	**Choices**	**Correct Answer**
**1**	During the pre-brief, the facilitator should remind participants how to manage complications.	True/False	False
**2**	During the pre-brief, the facilitator should allow providers time to review the simulation area.	True/False	True
**3**	The pre-brief is part of making a safe learning space.	True/False	True
**4**	A participant can ask the facilitator for clinical help at any time during a simulation.	True/False	False
**5**	Identify the 4 steps of the pre-brief.	1. Debrief huddle<br>2. Tour of the simulation and supplies<br>3. Rules of simulation<br>4. Obstetric and newborn care<br>5. Things to remember during simulation	2. Tour of the simulation and supplies<br>3. Rules of simulation<br>5. Things to remember during simulation<br>6. Communication techniques and RMC components

		6. Communication techniques and RMC components<br>7. History of simulation	
**Module 5 Survey Tool: Super Divya defends the Pre-brief**			
**S. No.**	**Knowledge Questions**	**Choices**	**Correct Answer**
**1**	A genuine self-practice can help a facilitator calm her mind to feel centered.	True/False	True
**2**	For a successful simulation training, the facilitators need to ignore their own emotions.	True/False	False
**3**	The facilitator spray is an imaginary tool that can be used to:	1. Help recognize participants emotions<br>2. Refresh the air of the simulation area<br>3. Help recognize facilitators emotions<br>4. Repel the bugs of the simulation area	5. Help recognize facilitators emotions
**Acceptability, Adoption and Feasibility:**			
**Acceptability Questions**	1. Did you like viewing Super Divya modules?<br>2. Do you feel good about using Super Divya?<br>3. Did you enjoy learning with the Super Divya?<br>4. Do you feel that the skills presented by Super Divya will be useful in facilitating future simulation trainings?<br>5. How satisfied are you with Super Divya platform itself?<br>6. Is the Super Divya story clear?		**Choices:**<br><br>**a)** Not at all<br>**b)** A little bit<br>**c)** A lot
**Adoption Questions**	1. Have you discussed with others (e.g. family, friends, coworkers) your experiences with Super Divya?<br>2. Would you use the Super Divya for training in the future? (Only for NMS)<br>3. Will using the Super Divya be a high priority in the future? (Only for NMS)		**Choices:**<br><br>**a)** Not at all<br>**b)** A little bit<br>**c)** A lot
**Feasibility Questions**	1. Is it easy for you to access and navigate the Super Divya modules?<br>2. Do you have enough time to watch the Super Divya modules?<br>3. Are you supported enough to participate in Super Divya modules?<br>4. Do you have the resources (data / technology) to watch Super Divya?		**Choices:**<br><br>**a)** Not at all<br>**b)** A little bit<br>**c)** A lot

Module 6 Survey Tool: Teamwork and communication part 1			
**S. No.**	**Knowledge Questions**	**Choices**	**Correct Answer**
**1**	Empathy means to understand one's and other's feelings?	True/False	True
**2**	One of the goals of the debrief huddle is to decide which nurses should speak up first.	True/False	False
**3**	When should a facilitator conduct a debrief huddle?	1. Prior to a simulation<br>2. During the simulation<br>3. Immediately after the simulation<br>4. Neither necessary nor an important step of simulation	3. Immediately after the simulation
**4**	The debrief huddle helps in:	1. Understanding one's own and the participants' feelings during the day<br>2. Understanding one's own and the participants feelings about the simulation and set objectives for the debrief<br>3. Identify which providers need additional training<br>4. Setting objectives for the next simulation	2. Understanding one's own and the participants feelings about the simulation and set objectives for the debrief
**5**	What should a simulation facilitator do during the release moment?	1. Disperse the group and let them do their routine duties<br>2. Let the providers take a snack break	4. Carefully listen and watch the providers

		3. Start taking notes about what went well and what could be done better<br>4. Carefully listen and watch the providers	
**6**	Empathy goggles help a facilitator identify the nurses who know the right answers during the debrief.	True/False	False
